# Development of an *Escherichia coli* Cell-Based Biosensor for Aspirin Monitoring by Genetic Engineering of MarR

**DOI:** 10.3390/bios14110547

**Published:** 2024-11-12

**Authors:** Yeonhong Kim, Yangwon Jeon, Kyeoungseok Song, Haekang Ji, Soon-Jin Hwang, Youngdae Yoon

**Affiliations:** Department of Environmental Health Science, Konkuk University, Seoul 05029, Republic of Korea; k99yh@konkuk.ac.kr (Y.K.); ywjun69@konkuk.ac.kr (Y.J.);

**Keywords:** whole-cell biosensor, transcription factor (TF), *mar* operon, aspirin, genetic engineering

## Abstract

Multiple antibiotic resistance regulators (MarRs) control the transcription of genes in the *mar* operon of *Escherichia coli* in the presence of salicylic acid (SA). The interaction with SA induces conformational changes in the MarR released from the promoter of the *mar* operon, turning on transcription. We constructed an SA-specific *E. coli* cell-based biosensor by fusing the promoter of the *mar* operon (P*_marO_*) and the gene that encodes an enhanced green fluorescent protein (*egfp*). Because SA and aspirin are structurally similar, a biosensor for monitoring aspirin can be obtained by genetically engineering MarR to be aspirin (ASP)-responsive. To shift the selectivity of MarR toward ASP, we changed the residues around the ligand-binding sites by site-directed mutagenesis. We examined the effects of genetic engineering on MarR by introducing MarRs with P*_marO_*-*egfp* into *E. coli*. Among the tested mutants, MarR T72A improved the ASP responses by approximately 3 times compared to the wild-type MarR, while still showing an SA response. Although the MarR T72A biosensor exhibited mutual interference between SA and ASP, it accurately determined the ASP concentration in spiked water and medicine samples with over 90% accuracy. While the ASP biosensors still require improvement, our results provide valuable insights for developing *E. coli* cell-based biosensors for ASP and transcription factor-based biosensors in general.

## 1. Introduction

Environmental systems are critical to human health; therefore, contamination caused by anthropogenic activities is monitored and controlled [1,2,3]. Environmental pollution poses potential risks to human health. Therefore, diverse contaminants, such as heavy metals, pesticides, and chemicals originating from industry, are strictly controlled and regulated [4,5,6]. However, other sources of environmental pollution have emerged with rapid industrial development. These include antibiotics used in agriculture and pharmaceuticals [7,8,9]. The risks posed by antibiotics and chemicals from drugs have been intensively investigated, and they are strictly regulated against. However, the widespread use of these chemicals and the emergence of new products have increased the risk to human health.

Some drugs are not harmful to the environment because they are present in extremely low concentrations, whereas others can be detrimental [9]. Although harmful drugs are controlled and regulated by authorities, pharmaceuticals are sometimes emitted from drug manufacturing and the disposal of expired drugs [10,11]. There are many reports on the impact of drug pollution on the environment, including aquatic systems and soils [12,13]. Adverse effects on microbial communities and aquatic organisms in the environment are the primary impacts; however, the impacts of drug pollution are not restricted to environmental systems. Similar to other pollutants, pharmaceuticals are destined for eventual destruction via diverse routes. Therefore, it would be prudent to regulate their use, production, and disposal.

Aspirin (ASP), which is also known as acetylsalicylic acid, is one of the most commonly used pharmaceuticals worldwide. It was synthesized in the 19th century and used to treat inflammation, pain, and cardiovascular diseases [14]. ASP has also been reported to exhibit anti-inflammatory, anti-platelet, anti-cancer, and anti-microbial activities [15,16,17]. Consequently, the use of ASP has continued to increase, and millions of people consume it daily. However, the release of pharmaceuticals, including ASP, into environmental systems is considered a significant issue. Pharmaceuticals, including ASP, have been detected in both wastewater and freshwater systems [18,19,20,21]. In these studies, researchers have focused on the removal and detection of chemicals originating from drugs. Moreover, it has been shown that ASP has ecotoxic effects on the biological functions of organisms in environmental systems [22,23]. Therefore, it is crucial to minimize the influx of pharmaceuticals into the environment to avoid potential risks.

To minimize the potential risks posed by drugs, it is necessary to develop techniques to detect, monitor, and remove pharmaceuticals, including ASP. There is widespread awareness of the potential threats posed by pharmaceuticals to environmental systems. Therefore, there have been numerous studies on the detection and monitoring of these chemicals using analytical instruments, such as high-performance liquid chromatography (HPLC) and mass spectroscopy (MS) [24,25]. Moreover, chemical sensors have been used as platforms for detecting those pharmaceuticals with rapid advances in nanomaterial- and nanofabrication technologies. By integrating nanomaterial-based target sensing elements on electrodes, the chemical sensors were able to detect pharmaceuticals [26,27,28]. Nonetheless, the chemical sensors were also based on analytical instruments such as cyclic voltammetry, electrochemical impedance spectroscopy, and cyclic voltammetry. The instrument-based analysis is precise, accurate, and sensitive. However, this may be disadvantageous in terms of cost, time, and operational expertise. Therefore, there is a demand for new tools for simple, fast, and precise analysis.

Among these new tools, bacterial cell-based biosensors have been actively investigated because they are simple, inexpensive, and enable rapid analysis [29,30]. Cell-based biosensors employ sensing elements to recognize various targets. Most bacterial cell-based biosensors employ transcription factors (TFs) as sensing elements, which determine target selectivity and specificity [31,32]. The interaction between targets and TF induces the expression of genes that encode enzymes or fluorescent proteins acting as signal-reporting elements. Since living organisms are used as sensor platforms, the stability and regeneration of biomolecules playing sensing and signal reporting elements were not problematic issues. On the other hand, while the long-term storage and activity of biosensor cells might be a concern, the biosensor cells can be stored as stocks for extended periods and regenerated by plasmid transformation at any time without losing their activity. Additionally, like other sensor systems, these types of biosensors require analytical instruments such as UV spectroscopy and fluorescence spectroscopy, depending on the type of output signals. This could be seen as a disadvantage, but it is inherent to biosensors based on living cells. Nonetheless, it would be pivotal to construct new cell-based biosensors as well as to solve the potential disadvantages to enhance the application of biosensors.

In the present study, we constructed *Escherichia coli* cell-based biosensors for ASP monitoring based on the *mar* operon. MarR is a protein that regulates the *mar* operon. It is known to interact with SA [33], and its target selectivity and specificity can be modulated by genetic engineering. We obtained an ASP-interacting MarR mutant by genetic engineering. A biosensor employing the selected MarR mutant demonstrated an enhanced response to ASP. We also investigated the practical applications of the new biosensor by quantifying the amount of ASP in artificially amended water samples and aspirin tablets. Although further studies are needed to improve the performance of the ASP biosensor, it is applicable to the monitoring of ASP in environmental systems. Moreover, a strategy for generating new biosensors from existing genetic systems would be helpful to scientists working in this area of research.

## 2. Materials and Methods

### 2.1. Materials

*E. coli* BL21(DE3) and DH5α were used as competent cells for gene cloning and host cells for biosensors, respectively. The Quick and Easy *E. coli* Gene Deletion Kit (Gene Bridges, Heidelberg, Germany) was used to delete the endogenous *marR* in *E. coli* BL21. Enzymes, including restriction enzymes and ligases, were purchased from Takara (Kusatsu, Shiga, Japan). Hotstar Taq polymerase (Qiagen, Hilden, Germany) was used for gene amplification and Turbo Pfu (Invitrogen, Waltham, MA, USA) was used for site-directed mutagenesis. SA, ASP, ferulic acid (FER), *p*-coumaric acid (*p*-COU), caffeic acid (CAF), methyl salicylate (MES), 3,4-dihydroxy benzoic acid (3,4-DHB), benzoic acid (BA), 3-chlorobenzoic acid (3-CHB), and 3-hydroxybenzoic acid (3-HB) were purchased from Sigma Aldrich (Steinheim, Germany) and prepared as 50 mM stock solutions in dimethyl sulfoxide (DMSO). Commercial aspirin tablets, Aspirin Protect (Bayer, Leverkusen, Germany), were purchased and used for the quantification. The primers used in the present study were synthesized by Macrogen (Seoul, Republic of Korea), and all DNA sequences were confirmed by sequencing. The primers used for the genetic engineering of MarR are listed in Supplementary Table S1.

### 2.2. Construction of Plasmids and Biosensors

The reporter plasmid pMarO-eGFP, consisting of the promoter region of the *mar* operon and *egfp*, was constructed as described previously [34]. The promoter region was inserted into pET15(a) with BglII/XbaI to replace the T7 promoter, and *egfp* was inserted downstream of the promoter region with BamHI/XhoI. *marR* was amplified via polymerase chain reaction and inserted into the pCDF-duet with NcoI/NotI to generate wild-type (WT) pCDF-MarR. Based on the 3-D structure of MarR, the residues around the SA-binding sites 1 and 2 were targeted for the genetic engineering of MarR. MarR mutants were obtained from pCDF-MarR WT via site-directed mutagenesis using an appropriate primer pair. Single, double, and triple mutations in MarR were generated by a combination of mutagenesis techniques. Each mutant was co-transformed with a reporter plasmid into *marR*-deficient *E. coli* BL21 (*E. coli-∆marR*) to generate a biosensor, which was used in an assay to evaluate the effects of genetic engineering on target selectivity and sensitivity.

### 2.3. Structural Analysis of MarR

The 3-D structure of MarR has been solved and deposited in the Protein Data Bank (PDB ID numbers 1JGS and 5H3R) [35,36]. The structure was visualized and analyzed using the PyMol software package (version 3.1). MarR forms a homodimer, and each monomer has two SA-binding sites: binding site 1 faces DNA and the other site is located opposite to binding site 1. Based on structural analysis, the amino acids involved in SA binding and located close to SA were selected for mutagenesis. The residues Gln42, Met74, and Arg77 in SA-binding site 1; Leu68, Thr72, Arg86, and Val96 in SA-binding site 2; and Leu33, Val84, and Leu100 located close to the SA-binding sites were targeted for genetic engineering.

### 2.4. Biosensor Assays

The biosensors generated by the co-transformation of pCDF-MarRs and pMarO-eGFP into *E. coli*-*marR* were cultured overnight at 37 °C. For the biosensor assays, the overnight cultures were inoculated into fresh Luria–Bertani (LB) broth until the optical density at 600 nm (OD_600_) reached 0.5–0.6. To test target selectivity, the cells were exposed to 1 mM concentrations of SA, ASP, FER, *p*-COU, CAF, MES, 3,4-DHB, BA, 3-CHB, and 3-HB, and their fluorescence intensities were measured after 0.5, 1, and 2 h. A BioTek Synergy LX multimode microplate reader (Agilent BioTek, Winooski, VT, USA) was used to measure the fluorescence intensity of eGFP at excitation/emission wavelengths of 480/510 nm. After screening for target selectivity, the selected biosensors were used in a biosensor assay with 0–2 mM SA and ASP. The responses to the chemicals are represented as induction coefficient (IC) values, defined as [arbitrary unit of eGFP with exposure/arbitrary unit of eGFP without exposure]. The data were obtained from more than three experiments using different batches of biosensor cells and indicated as mean values with standard deviations.

### 2.5. Quantification of Aspirin by Biosensors

To evaluate its applicability, the concentrations of ASP in artificially amended water samples and aspirin tablets were evaluated. Artificially amended samples with concentrations of 10, 20, and 50 mM were prepared and subsequently investigated using biosensors. The 20.0 mg of aspirin tablets were ground, dissolved in 1 mL of ethanol, and applied to the biosensors. Among the tested biosensors, MarR T72A, which demonstrated an enhanced ASP response, was selected for aspirin quantification. As described above, the biosensor cells were freshly incubated in LB broth and then exposed to 0–1 mM ASP until the OD_600_ reached 0.4. After exposure for 2 h, the intensities of the fluorescence signals were measured and converted to IC values. The IC values were then plotted against the ASP concentration to construct standard quantification curves. The aspirin concentrations in the artificially amended samples and tablets were determined based on the standard curves.

### 2.6. Data Analysis

All experimental data were obtained from more than three tests, and the values are indicated as the mean and standard deviation. Statistical analysis and data validation were performed using the R version 4.3.0 package DescTools version 0.99.59 [37,38]. Significant differences in the data compared to the control were validated using Dunnett’s test.

## 3. Results

### 3.1. Characteristics of the Mar Operon System-Based Biosensors

**Figure 1:**
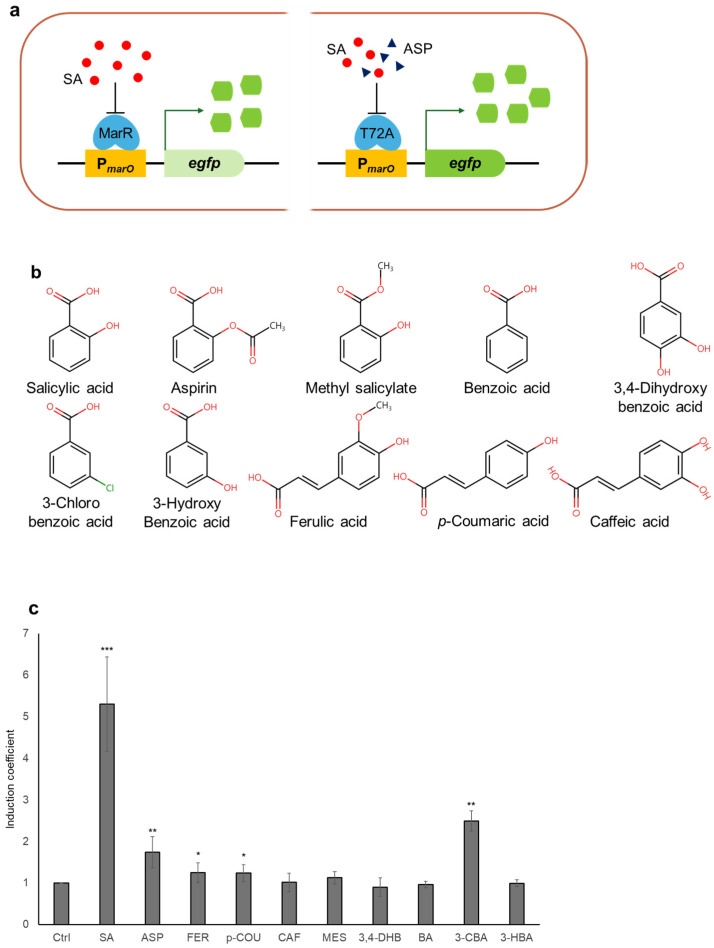
*Escherichia coli* cell-based biosensors based on the *mar* operon system. (**a**) The diagram of the working mechanism of biosensors based on the *mar* operon system. (**b**) Structures of the chemicals tested in the present study. (**c**) Responses of the biosensor comprising MarR WT to the various chemicals. The values are indicated as induction coefficient values and standard deviations. The data were replicated more than three times and asterisks indicate significant differences in data compared to the control (* *p* ≤ 0.05, ** *p* ≤ 0.01, *** *p* ≤ 0.001 using Dunnett’s test).

The *E. coli* cell-based biosensors exploiting the *mar* operon system responded to SA. Because MarR is a transcription factor that regulates genes in the *mar* operon, the transcription of *egfp* under the operator region of the *mar* operon (P*_marO_*) was activated in the presence of SA (Figure 1a). Therefore, *E. coli* BL21 with WT MarR and pMarO-eGFP can be used in SA-specific biosensors, as reported previously [34]. Unlike in previous research, recombinant MarR WT and pMarO-eGFP were introduced into *marR*-deficient *E. coli* BL21 (*E. coli*-*marR*) to generate biosensors. Although MarR is known to interact with SA, it is necessary to verify its target selectivity with other chemicals with structural similarities. In the present study, 10 chemicals, including SA and ASP, were selected to verify the target selectivity of biosensors comprising MarR WT (Figure 1b). To validate the characteristics of the biosensors employing the *mar* operon system, the *E. coli* cell-based biosensors comprising MarR WT and pMarO-eGFP were exposed to 1 mM concentrations of these chemicals. After 2 h of exposure, the fluorescence intensities of eGFP induced by the chemicals were measured using a microplate reader, and the responses were indicated as IC values (Figure 1c). As expected, the biosensor responded most strongly to SA, with an IC value of approximately 5. In contrast, with the exception of 3-CHA, all the other chemicals produced IC values of <2. This revealed that MarR has superior target specificity to SA.

### 3.2. Genetic Engineering of MarR

**Figure 2:**
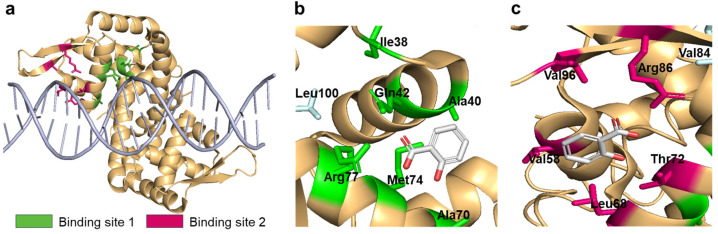
3-D structure of the MarR homodimer and SA binding sites. (**a**) Structure of the MarR WT dimer with DNA. Binding sites 1 and 2 are indicated in green and purple, respectively. (**b**) SA binding site 1 with residues involved in the SA interaction. (**c**) SA binding site 2 with residues involved in SA binding.

As described above, the biosensor comprising MarR WT and pMarO-eGFP was SA-specific. Because the specificity of TF-based biosensors is determined by the TF (i.e., MarR in the present case), the target of the biosensors can be changed by modulating the specificity of the TF. Therefore, it is possible to shift the target specificity of biosensors from SA to other molecules possessing structural similarity, such as ASP, by genetic engineering using MarR. As shown in Figure 2, MarR acts as a TF by forming a homodimer that interacts with DNA [35]. The residues at the two SA-binding sites (i.e., sites 1 and 2) on each monomer are indicated in green and purple, respectively (Figure 2a). The residues involved in SA binding are depicted in Figure 2b,c and were selected as targets for mutagenesis. pCDF-MarR WT was used as a template, and a pair of primers was used for site-directed mutagenesis. The residues in binding site 1 (i.e., Ile38, Ala41, Glu42, Ala70, Met74, and Arg77) were changed to other amino acids to modulate charge, hydrophobicity, and bulkiness. Val58, Leu68, Thr72, Arg86, and Val96 residues at binding site 2 were targeted for genetic engineering. Double and triple mutants were generated because there are two different SA-binding sites. All mutants were introduced into *E. coli-marR* with pMarO-eGFP and subjected to a biosensor assay to evaluate their sensing properties. The engineered MarRs used in the present study are listed in Table 1 and Supplementary Table S2 along with the ICs for SA and ASP. We noticed that binding site 1 faces the DNA and the other site is located on the opposite side. Although the four SA molecules were co-crystallized with the MarR homodimer, SA docking to binding site 1 interfered with the interaction of MarR with DNA.

### 3.3. Effects of MarR Engineering on the Characteristics of the E. coli Cell-Based Biosensors

**Figure 3:**
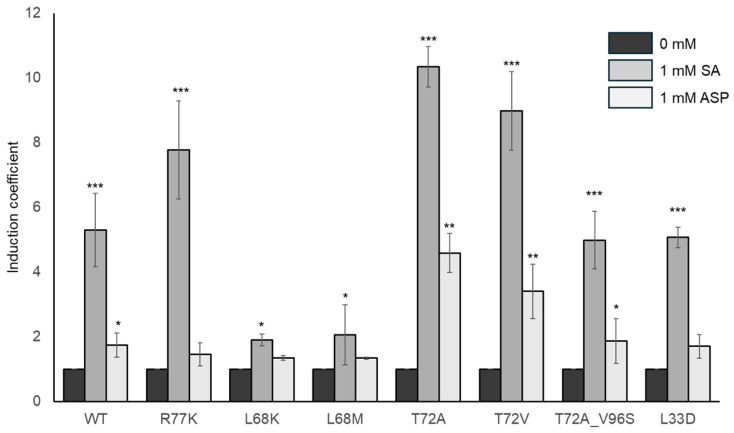
Responses to the SA and ASP of biosensors based on engineered MarRs. Induction coefficient values of biosensors comprising MarR WT and the seven mutants with 1 mM SA or ASP. Data are presented as mean values and standard deviations from more than three replicated experiments. Asterisks indicate significant differences in the data compared to the control (* *p* < 0.05, ** *p* < 0.01, *** *p* < 0.001 using Dunnett’s test).

To evaluate the effects of genetic engineering on MarR, all the mutants were introduced with pMarO-eGFP into *E. coli* to generate biosensors. Each biosensor was subjected to a biosensor assay to determine its responses to SA and ASP. The cells were exposed to each chemical (1 mM), and the intensities of the resulting fluorescence signals were measured after 2 h. Among the many MarR mutants, those with mutations at Ile38, Ala41, Ala70, Val58, and Arg77 produced no fluorescence signals, whereas the others produced strong background fluorescence (Supplementary Table S2). The former result may have arisen because the engineered MarRs were not released from the promoter region, which suppressed the transcription of *egfp*. Strong background signals may have been caused by the weakening of the interaction between the MarRs and the promoter regions. Although the engineered MarRs responded to SA and ASP, their IC values toward SA and ASP were lower than those of MarR WT. Engineered MarRs with fewer than two ICs were excluded from further investigation. MarRs that significantly affected the SA and ASP responses were investigated further. As shown in Figure 3, the engineered MarRs comprising Arg77 and Thr72 exhibited modulated responses compared to MarR WT. Arg77Lys (R77K) exhibited an increased response to SA compared to the WT, but its response to ASP was slightly decreased. The Thr72Ala (T72A) and Thr72Val (T72V) mutants exhibited increased responses to SA and ASP, whereas L68 lost those responses. This may be because the mutations abolished the hydrogen bond between Thr72 and the 2-hydroxyl group of SA, thereby facilitating the entrance of ASP into the binding site. In the case of double mutagenesis, MarR T72A/V96S, with mutations at both SA-binding sites, exhibited similar levels of response to those of MarR WT. Herein, it was difficult to obtain ASP-specific biosensors based on the genetic engineering of MarR, and it might be reasoned that MarR has two distinct SA-binding sites. Nonetheless, the mutation at Thr72 in MarR produced an enhanced ASP response, which implied the possibility of biosensors for monitoring. Therefore, MarR T72A and T72V, which exhibited strong ASP responses, were selected as biosensor elements, and their applicability to ASP monitoring was evaluated in further investigations. The responses of the biosensors to SA and ASP shown in Figure 3 are summarized in Table 1 as IC values with background signals.

### 3.4. Response to the ASP of the Biosensors with Engineered MarRs

**Figure 4:**
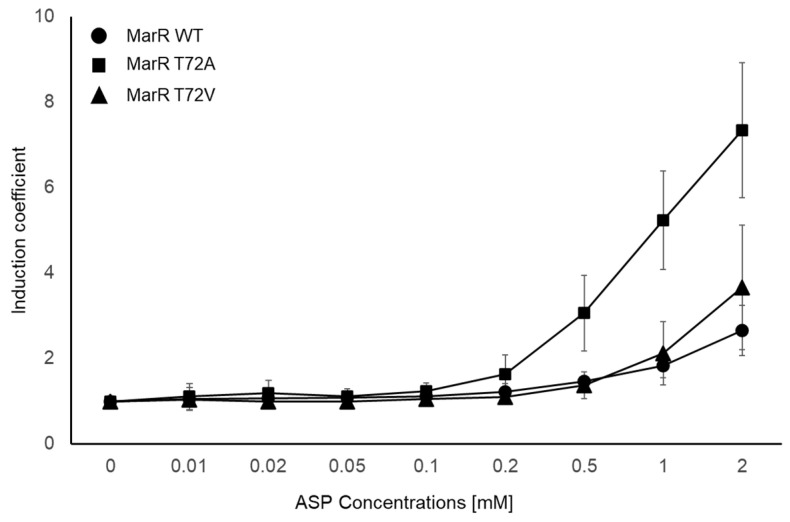
Responses of selected biosensors to 0–2 mM ASP. Biosensors comprising MarR WT, T72A, and T72V as sensing elements were exposed to ASP, and the responses are indicated as induction coefficient values. The data are indicated as mean values and standard deviations from more than three replicated experiments.

Since the biosensors with MarR T72A and T72V showed enhanced responses to ASP, they were selected as sensing elements for evaluating ASP specificity. Prior to the assay, it was necessary to validate the selectivity of the biosensors, which were then subjected to selectivity tests with 1 mM of SA and its derivatives. As shown in Figure 1c, the biosensors employing MarR T72A and T72V as sensing elements responded to both SA and ASP, with no significant response to the other compounds (Supplementary Figure S1). Then, biosensors with MarR WT, T72A, and T72V were exposed to 0–2 mM ASP for 2 h, and the fluorescence intensities were measured to evaluate the effects of engineered MarRs on aspirin specificity. As shown in Figure 4, the intensities of the signals produced by the biosensors increased in a concentration-dependent manner, and MarR T72A had an IC of approximately 7 when exposed to 2 mM ASP. In contrast, the response of T72V to ASP was similar to that of MarR WT and was not significantly enhanced. Based on these results, it was concluded that biosensors with MarR T72A could be used for ASP monitoring and quantification. Although the biosensor comprising MarR T72A responded to ASP with significant IC values, it was not ASP-specific. Therefore, it is necessary to validate the interference of SA with ASP detection before using the biosensor for ASP quantification.

### 3.5. Effects of SA on ASP Monitoring by E. coli Cell-Based Biosensors

**Figure 5:**
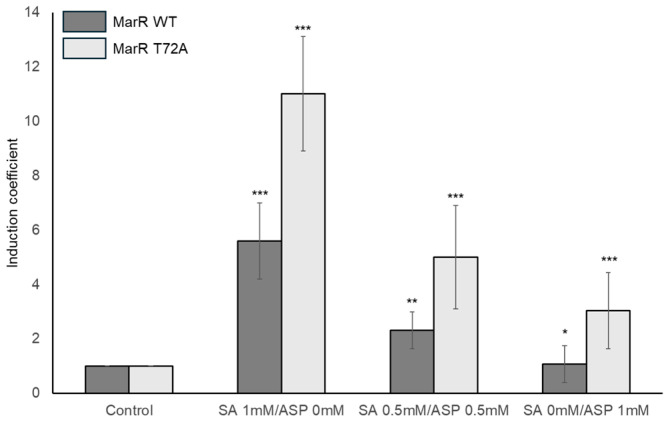
Responses of biosensors to SA and ASP. The biosensors comprising MarR WT and T72A were exposed to SA or ASP (1 mM) and both SA and ASP (0.5 mM). The signals caused by chemical exposure are indicated by induction coefficient values. The data are represented as mean values and standard deviations from more than three replicated experiments. Asterisks indicate significant differences in data compared to the control (* *p* ≤ 0.05, ** *p* ≤ 0.01, *** *p* ≤ 0.001 using Dunnett’s test).

As described above, it is difficult to generate ASP-specific MarRs by genetic engineering based on 3-D structural analysis. Although the target specificity of *E. coli* cell-based biosensors has been modulated by the genetic engineering of MarR, the biosensor comprising MarR T72A responded to both SA and ASP. The effects of SA on ASP monitoring should be validated before this biosensor is used for ASP quantification. To examine this issue, biosensors comprising MarR WT and T72A were exposed to 1 mM SA, 0.5 mM SA/ASP, and 1 mM ASP, and the responses were determined after 2 h. As shown in Figure 5, the biosensor comprising MarR WT only responded to SA, whereas MarR T72A responded to both SA and ASP. Moreover, the signals produced by the biosensor comprising MarR T72A were stronger when it was exposed to 0.5 mM SA/ASP than when it was exposed to 1.0 mM ASP. Therefore, we conclude that the new biosensor comprising MarR T72A has dual specificity SA and ASP, and SA is preferred. Although the biosensor based on MarR T72A and pMarO-eGFP responded to both SA and ASP, it could be used to quantify ASP in the absence of SA. However, the specificity of the biosensor for both compounds is not ideal for ASP quantification and requires improvement.

### 3.6. Aspirin Quantification Using E. coli Cell-Based Biosensors

**Figure 6:**
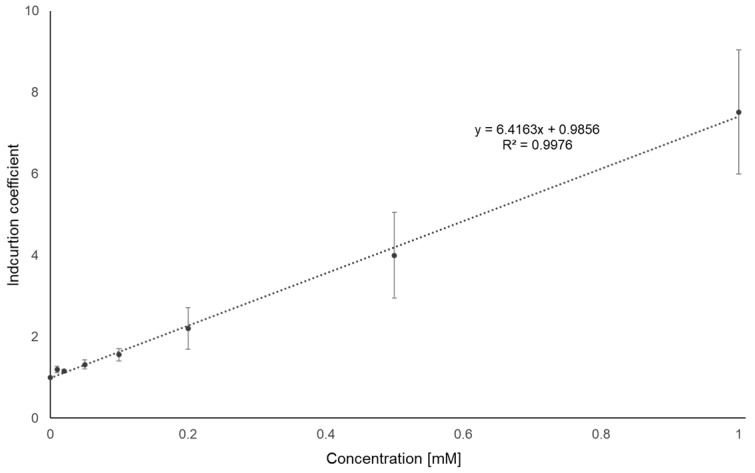
Standard curves for ASP quantification. Biosensors comprising MarR T72A were exposed to 0–1 mM ASP for 2 h, and the fluorescence signals obtained are represented by induction coefficient values. The data and standard deviations were obtained from three replicated experiments.

To examine the applicability of MarR T72A, we determined the amount of ASP in artificially contaminated water samples and commercially available aspirin tablets from pharmacies. The samples were supplied to the biosensor cells at several dilutions, and a known concentration of ASP was supplied to the biosensor cells to construct a standard curve for quantification. Following the same procedure used for the biosensor assay, the fluorescence signals induced by ASP were determined after 2 h of exposure. As shown in Figure 6, the standard curve was obtained by plotting the concentration of ASP (0–1 mM) against the IC values. Since 0.01 mM was the lowest concentration of ASP showing a significant difference, the detection limit of this biosensor would be 0.01 mM. A linear regression fitting yielded an R^2^ value of 0.9976; therefore, the equation was used for the quantification of ASP in the samples. The concentrations of the tested samples were determined using this equation, and the results are listed in Table 2. With regard to the water samples, the biosensor indicated 9.53 ± 0.49, 21.52 ± 3.14, and 47.86 ± 2.98 mM for the 10, 20, and 50 mM samples, respectively, so the biosensor assay accuracy was over 93%.

In the case of ASP quantification in aspirin tablets, the biosensor indicated that the amount of ASP was 31.68 ± 1.25, which was much lower than the expected value estimated from the description of ASP contents. To confirm the performance of the new biosensor for ASP quantification, the aspirin tablet samples were subjected to liquid chromatography with tandem mass spectrometry (LC-MS-MS) analysis. The results are listed in Table 2 and the concentrations of ASP in the prepared samples were determined to be 33.8 ± 0.5 mM by LC-MS-MS. In conclusion, the results revealed that the new biosensor was reliable compared to instrumental analysis, suggesting its potential as an alternative tool for ASP quantification.

## 4. Discussion

In the present study, we aimed to generate ASP-sensing *E. coli* cell-based biosensors via genetic engineering using MarR [33]. Because MarR is a regulatory protein that controls the *mar* operon in *E. coli* upon interaction with SA, we constructed an *E. coli* biosensor comprising MarR/P*_marO_*, as investigated in a previous study [34]. Although the biosensor based on MarR WT exhibited a slight response to ASP, as shown in Figure 1c, it was insufficient for use as a sensor for ASP monitoring. As shown in Figure 2, MarR has two SA-binding sites in each monomer, but site 1 faces the DNA side. Owing to this structural alignment, engineered MarRs with mutations at site 1 produced strong fluorescence signals without chemical exposure and no response to chemicals (Supplementary Table S2). These mutations may interfere with the DNA binding of MarRs, thereby abolishing the role of repressors in *E. coli* biosensors. In contrast, the genetic engineering of SA-binding site 2 resulted in improved properties, as shown in Figure 3. The R77K, T72A, and T72V mutants exhibited increased SA responses, whereas the others showed insignificant responses compared to the WT. Among the 38 MarR mutants, T72A and T72V exhibited enhanced responses to ASP. Although they responded to SA more strongly than to ASP, they probably provided the best ASP response generated by MarR. Because ASP and SA share structural similarities, it is difficult to obtain ASP-specific MarRs by genetic engineering. In common with other studies on cell-based biosensors, the present study demonstrated multiple target responses toward SA and ASP. For example, Truan et. al. reported that *E. coli* cell-based biosensors using HbaR from *Rhodopseudomonas palustris* responded to benzoic acid, SA, and ASP [39]. Chen et al. reported that biosensors employing SalR in *Acinetobacter baylyi* ADP1 and a P*_sal_* promoter responded to multiple chemicals, including SA and ASP [40]. Moreover, Monteiro et. al. also reported that biosensors employing genetically engineered BenR exhibited modulation of target specificity and responded to multiple targets, including ASP [41]. An ASP-specific biosensing system would be preferable. However, we believe that the biosensor obtained in the present study is better-suited to the monitoring of ASP than the sensors described in previous studies.

Specificity toward SA should be carefully considered when developing a new biosensor for ASP monitoring. Although extensive genetic engineering was applied to obtain MarR mutants specific to ASP, achieving this was challenging due to inherent characteristics of MarR, such as its SA specificity and the presence of two SA binding sites. As shown in Figure 5, the performance of the *E. coli* cell-based biosensor containing MarR T72A for ASP detection was compromised by exposure to SA. Because of dual responses to SA and ASP, the performance of the new biosensors for ASP monitoring could be compromised by mutual interference by SA. Further investigation will be needed to overcome this issue, ensuring accurate ASP quantification and expanding the application range of these new biosensors. However, this limitation could be partially addressed at this stage by using both a biosensor with MarR WT and one with the T72A mutant. Since the WT biosensor responds only to SA, interference from SA in the quantification of SA and ASP mixtures was corrected by using both biosensors. Although this approach might be optimal, we did not use both biosensors to quantify SA and ASP mixtures in the current study. Instead, we evaluated the performance of the new biosensor for ASP detection based on its quantification accuracy. As a result, it demonstrated over 90% accuracy in quantifying ASP in artificially amended water samples and aspirin tablet samples. Although a biosensor specific to ASP alone was not achieved through genetic engineering in this study, the strategy presented here will aid in developing new biosensors using existing genetic systems, thus expanding the potential of biosensors for practical applications.

## 5. Conclusions

This study successfully developed an *E. coli* cell-based biosensor for aspirin (ASP) monitoring by engineering the MarR protein, originally responsive to salicylic acid (SA). Through site-directed mutagenesis, the MarR T72A variant was identified as a promising candidate, showing a threefold improvement in response to ASP compared to the wild-type MarR. This biosensor demonstrated over 90% accuracy in ASP quantification from spiked water and aspirin tablets, highlighting its potential for environmental and pharmaceutical monitoring applications. However, mutual interference between SA and ASP remains a significant challenge, as the biosensor lacks exclusive specificity to ASP, leading to dual responses that could compromise ASP quantification in environments where both substances are present. To address this, future work should focus on refining the biosensor’s specificity, potentially by further structural modifications to MarR or by pairing it with additional selective sensing mechanisms. Despite these limitations, this research provides valuable insights into the development of transcription factor-based biosensors, advancing the field of whole-cell biosensing for ASP and potentially other structurally similar contaminants.

## Figures and Tables

**Table 1:** Responses of biosensors with engineered MarRs to SA and ASP.

Site	Mutated Amino Acid	IC (1 mM)	FS
	WT	SA(5.3 ± 1.1)/ASP(1.7 ± 0.4)	369
Site 1	R77K	SA(7.8 ± 1.5)/ASP(1.5 ± 0.4)	1074
Site 2	L68K	SA(1.9 ± 0.2)/ASP(1.3 ± 0.1)	1484
	L68M	SA(2.1 ± 0.9)/ASP(1.3 ± 0.2)	1386
	T72A	SA(10.3 ± 0.6)/ASP(4.6 ± 0.6)	618
	T72V	SA(8.1 ± 0.3)/ASP(3.4 ± 0.8)	563
	T72A/V96S	SA(5.0 ± 0.3)/ASP(1.9 ± 0.7)	468
Other site	L33D	SA(5.1 ± 0.3)/ASP(1.7 ± 0.4)	378

MarR = multiple antibiotic resistance regulator; SA = salicylic acid; ASP = aspirin; IC = induction coefficient; FS = background fluorescence signal without chemical exposure.

**Table 2:** Quantification of ASP in artificially amended samples and in aspirin tablets.

		Determined Concentration [mM]		Accuracy (%)
Artificially amended samples	10 mM	9.53 ± 0.49		95.3
	20 mM	21.52 ± 3.14		92.9
	50 mM	47.86 ± 2.98		95.7
		Biosensors	LC-MS-MS	
Aspirin tablets		31.68 ± 1.25	33.8 ± 0.5	93.7

ASP = aspirin; LC-MS-MS = liquid chromatography with tandem mass spectrometry.

## Data Availability

Data are contained within the article.

## Supplementary Materials

The following supporting information can be downloaded at https://www.mdpi.com/article/10.3390/bios14110547/s1: Figure S1: The effects of engineered MarRs on selectivity of biosensors; Table S1: The list of primers used in this study; Table S2: The list of biosensors based engineered MarRs and responses to SA and ASP.
