# Supplementary material for: Development of an Escherichia coli Cell-Based Biosensor for Aspirin Monitoring by Genetic Engineering of MarR
Source: Biosensors (Basel). 2024 Nov 12;14(11):547. doi: 10.3390/bios14110547 (PMC11591804; doi:10.3390/bios14110547)
Supplement: Supplementary file 1 [file biosensors-14-00547-s001.zip › biosensors-3204547-supplementary.pdf]

Supplementary Table S1. The list of primers used in this study.

	**Sequences (5' to 3')**	**Restriction enzyme**	
WT	TACCATGGGCGTGAAAAGTACCAGCGATCTGTT	*NcoI*	Forward
	ATGCGGCCGCGGCCTTACGGCAGGACTTTCTTAA	*NotI*	Reverse
I38D	GTATCTGTCTCCGCTGGATGACACCGCGGCACAGTTTAAG		
	CTTAAACTGTGCCGCGGTGTCATCCAGCGGAGACAGATAC		
A41D	GGATATTACCGCGGACCAGTTTAAGGTGCTC		
	GAGCACCTTAAACTGGTCCGCGGTAATATCC		
A41E	GGATATTACCGCGGAGCAGTTTAAGGTGCTC		
	GAGCACCTTAAACTGCTCCGCGGTAATATCC		
A41H	GATATTACCGCGCACCAGTTTAAGGTG		
	CATACGGGTCAGATGTCCCAGGTCGAC		
A41R	CTGGATATTACCGCGCGACAGTTTAAGGTGC		
	GCACCTTAAACTGTCGCGCGGTAATATCCAG		
Q42A	GATATTACCGCGGCAGCATTTAAGGTGCTCTG		
	CAGAGCACCTTAAATGCTGCCGCGGTAATATC		
Q42E	GATATTACCGCGGCAGAGTTTAAGGTGCTC		
	GAGCACCTTAAACTCTGCCGCGGTAATATC		
L68K	GTATTGTCGGTCGACAAGGGAGCACTGACCC		
	GGGTCAGTGCTCCCTTGTCGACCGACAATAC		
A70H	GTCGACCTGGGACATCTGACCCGTATG		
	CATACGGGTCAGATGTCCCAGGTCGAC		
M74A	CACTGACCCGTGCACTGGATCGCCTG		
	CAGGCGATCCAGTGCACGGGTCAGTG		
R77A	CCCGTATGCTGGATGCCCTGGTCTGTAAAG		
	CTTTACAGACCAGGGCATCCAGCATACGGG		
R77G	CGTATGCTGGATGGACTGGTCTGTAAAG		
	CTTTACAGACCAGTCCATCCAGCATACG		
R77K	CGTATGCTGGATAAACTGGTCTGTAAAG		
	CTTTACAGACCAGTTTATCCAGCATACG		
R77Q	GTATGCTGGATCAGCTGGTCTGTAAAG		
	CTTTACAGACCAGCTGATCCAGCATAC		
L33D	CGATCCAGCATACGTACCAGTGCTCCCAGGTC		
	GTAATATCCAGCGGAGAGTCATACTCGTTAAGCAGG		
V58H	CGTGTATTACTCCGCATGAACTGAAAAAG		
	CTTTTTCAGTTCATGCGGAGTAATACACG		
V58K	GTGTATTACTCCGAAGGAACTGAAAAAGG		
	CCTTTTTCAGTTCCTTCGGAGTAATACAC		
T72A	CTGGGAGCACTGGCCCGTATGCTGG		
	CCAGCATACGGGCCAGTGCTCCCAG		
T72S	CCTGGGAGCACTGAGTCGTATGCTGGATC		
	GATCCAGCATACGACTCAGTGCTCCCAGG		
T72V	GACCTGGGAGCACTGGTACGTATGCTGGATCG		
	CGATCCAGCATACGTACCAGTGCTCCCAGGTC		
R86A	GCTGGGTGGAAGCATTGCCGAACCC		
	GGGTTCGGCAATGCTTCCACCCAGC		
V96S	GACAAGCGCGGCTCACTGGTAAAAC		
	GTTTTACCAGTGAGCCGCGCTTGTC		
V84D	CTGTAAAGGCTGGGACGAAAGGTTGCCGAAC		
	GTTCGGCAACCTTTCGTCCCAGCCTTTACAG		
L100D	CGTACTGGTAAAAGATACCACCGGCGGCG		
	CGCCGCCGGTGGTATCTTTTACCAGTACG		

Supplementary Table S2. The list of biosensors based engineered MarRs and responses to SA and ASP.

Site	MarRs	I.C (1mM)	F.S
Site 1	I38D	No response	-
	A41D	No response	-
	A41E	No response	-
	A41H	No response	-
	A41R	No response	-
	Q42A	SA(1.6±0.4); ASP(1.8±0.4)	4198
	Q42E	SA(1.4±0.1); ASP(1.2±0.0)	9854
	A70H	No response	-
	M74A	SA(1.4±0.1); ASP(1.5±0.2)	5576
	R77A	No response	-
	R77G	SA(1.2±0.0); ASP(1.3±0.3)	25317
	R77Q	SA(1.6±0.1); ASP(1.9±0.1)	5074
Site 2	V58H	No response	-
	V58K	No response	-
	T72S	No response	-
	R86A	SA(2.5±0.3); ASP(2.5±0.1)	3760
	V96S	No response	-
	T72A/R86A	SA(1.9± 0.2); ASP(2.0±0.3)	12540
Site 1&2	A41H/V58H	No response	-
	A41H/A70H	No response	-
	A41H/A70K	No response	-
	A41R/V58H	No response	-
	Q42A/T72A	SA(1.6±0.3); ASP(1.8±0.4)	12627
	Q42A/T72V	SA(1.5±0.4); ASP(1.4±0.1)	36721
	Q42A/R86A	No response	-
	R77K/T72A	SA(2.2±0.2); ASP(2.1±0.3)	3997
	R77Q/T72A	SA(1.5±0.3); ASP(1.7±0.3)	6288
	R77Q/T72V	No response	-
	Q42A/R77K/T72A	SA(1.5±0.4); ASP(1.5±0.1)	45703
Other sites	V84D	SA(1.1±0.3); ASP(1.2±0.2)	9023
	L100D	SA(1.0±0.1); ASP(1.3±0.2)	10981

[*] I.C: induction coefficient values; F.S: background fluorescence signal without chemical exposure

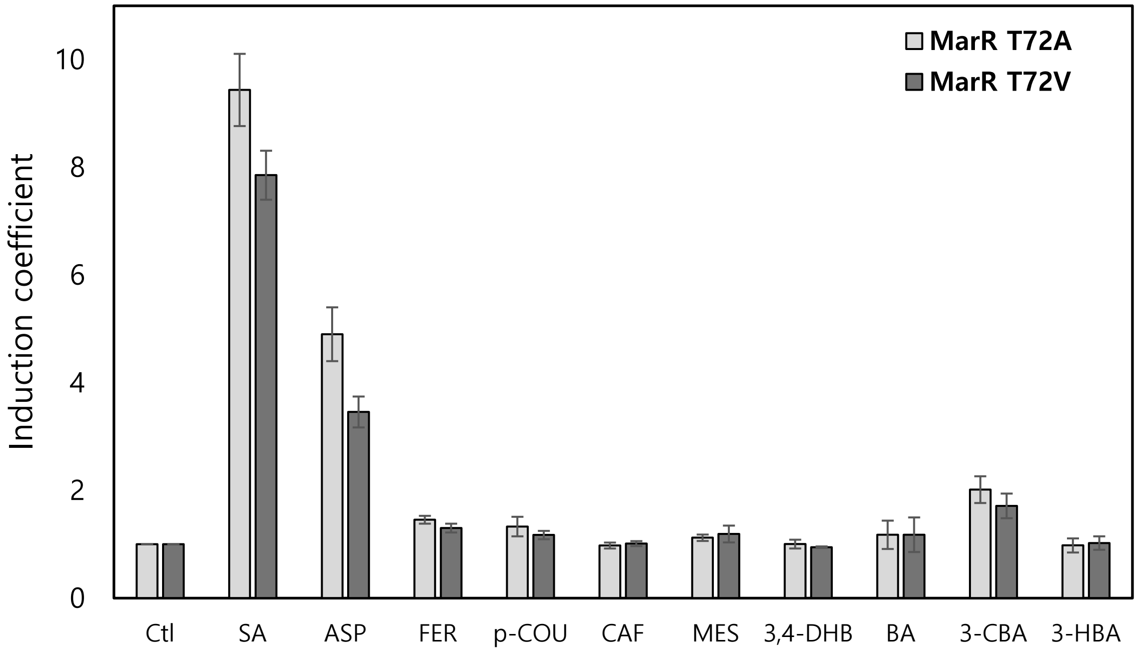

Supplementary Figure S1. The effects of engineered MarRs on selectivity of biosensors.

Responses of the biosensors based on MarR T72A and T72V to SA and other structurally similar chemicals are presented as induction coefficient values with corresponding standard deviations. The data were obtained from three independent experiments.
